# Methods to Improve the Solubility of Curcumin from Turmeric

**DOI:** 10.3390/life13010207

**Published:** 2023-01-11

**Authors:** Julia Górnicka, Martyna Mika, Oliwia Wróblewska, Paweł Siudem, Katarzyna Paradowska

**Affiliations:** 1 Student Research Group “Free Radicals”, Department of Organic and Physical Chemistry, Faculty of Pharmacy, Medical University of Warsaw, Banacha 1, 02-097 Warsaw, Poland; 2 Department of Organic and Physical Chemistry, Faculty of Pharmacy, Medical University of Warsaw, Banacha 1, 02-097 Warsaw, Poland

**Keywords:** curcumin, solubility, bioavailability, nanoparticles, cocrystals

## Abstract

Turmeric is a strong-taste component of spices characteristic of Indian cuisine. It is obtained from the turmeric rhizome (*Curcumae longae rhizoma*) and has been used for thousands of years not only for culinary purposes, but also for medicinal purposes. It contains a group of organic compounds called curcuminoids. Curcumin is the main representative of this group of compounds which is also most frequently studied. In recent years, bioactive curcuminoids (including curcumin in the first place) have become more and more popular due to a wide spectrum of their biological activity. The anticancer, antibacterial, anti-inflammatory, and antiaging effects of curcumin have been confirmed by numerous in vitro and in vivo studies, as well as in clinical trials. However, an obstacle to simple, clinical application of curcumin is its poor bioavailability (which is due to its hydrophobic nature) and its very weak water solubility. Therefore, many scientists are working on improving the solubility of curcumin in water, which is the topic of the present article. Attempts have been made to combine curcumin with nanoparticles (polysaccharide or silica). Nanosuspensions or complexes with cyclodextrins are also considered. A promising direction is the search for new polymorphic varieties as well as obtaining cocrystals with curcumin which are characterized by better water solubility.

## 1. Introduction

**Figure 1:**
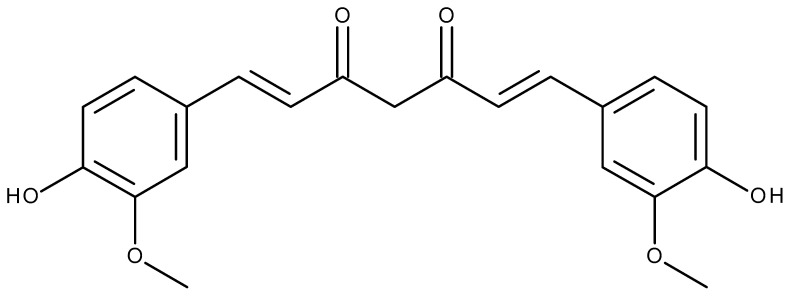
The structural formula of curcumin.

Curcuminoids are a group of organic compounds which are responsible for the yellow color of curcumin—a spice characteristic of Indian cuisine and obtained from the turmeric rhizome (*Curcumae longae rhizoma*). Curcumin is the main and at the same time best known and studied compound of this group. Curcumin is a dimeric derivative of ferulic acid (Figure 1). Some of its effects include the choleretic and anti-inflammatory effects.

Curcuminoids are well-known for their antioxidant [1], anti-inflammatory [2], antibacterial [3], antivirus, and even anticancer effects [4,5]. Numerous studies showed that curcumin has the potential to prevent multiple diseases, such as diabetes [6], Alzheimer’s disease [7,8], atherosclerosis [9], or neoplastic diseases, particularly those of the digestive tract [4]. Its immunomodulatory effect has been repeatedly demonstrated [10,11]. Curcumin was also found to have a notable impact on preventing osteoporosis as well as cardiovascular and liver diseases [5,12,13,14].

Curcumin itself arouses interest in three research directions: nutritional [15,16], pharmacological [17,18,19], and biological [20,21,22,23,24].

Unfortunately, curcumin is characterized by poor water solubility (0.6 µg/mL [25]), which also means low bioavailability (its level in the serum is up to 60 nM [26]). Due to the hydrophobic character of the curcumin molecule, this compound is practically not absorbed from the digestive tract. Moreover, a considerable part of curcumin undergoes inactivation in the process of liver metabolism. Its concentration in plasma does not exceed 1 μmol, and its highest concentration can be observed 1–2 h after consumption [26].

Multiple attempts have been made to increase the solubility of curcumin in water and its bioavailability because this compound could be a good alternative to many chemical substances, as it is safe to use, it is characterized by small toxicity, it has a great health-promoting potential, and it exerts multiple favorable effects on the human organism [27].

This article presents selected methods of increasing the water solubility of the main curcuminoid, i.e., curcumin.

## 2. Nanoparticles

**Figure 2:**
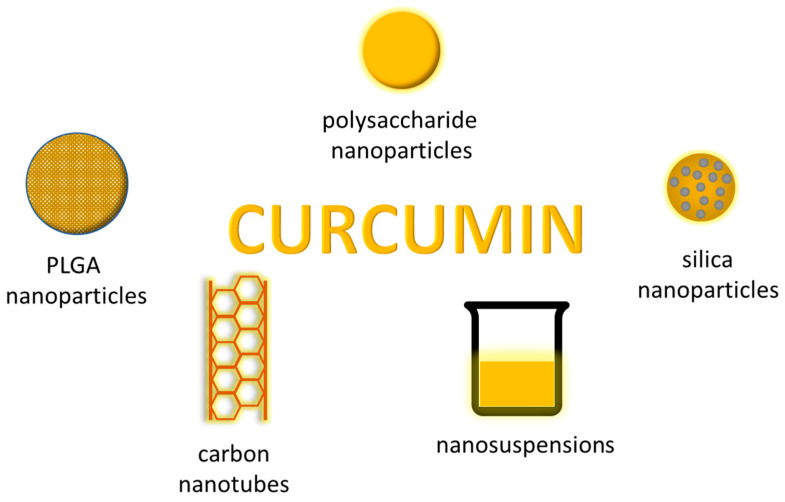
Selected nanoforms of curcumin used to improve its bioavailability.

Nanoparticles (NPs) are a broad class of materials that include particulate substances with one dimension smaller than or equal to 100 nm, which exist in the natural world, but also arise as a result of human activity. They have unique material properties, and the produced nanoparticles find practical applications in many fields, including medicine and pharmacy. The efficient production of nanoparticles is leading to exciting new drug delivery applications (Figure 2).

### 2.1. Encapsulation in Polysaccharide Nanoparticles

Nanoparticles are the particles of which at least one dimension is contained in the range below 100 nm. The investigated nanoparticles containing curcumin are fully biodegradable, biocompatible, and safe for the human organism [28]. These molecules are synthesized by the method of ionotropic gelation. It is a technique in which the polymer solutions undergo violent gelation in the presence of cations, and the course of this process depends on the concentration and valence of the cations. This mechanism allows one to dissolve curcumin in the water solution more effectively in comparison with the free fraction. The polymers used are chitosan, alginine, and carrageenan, which commonly occur in nature. They are used as carriers which are non-toxic for the human body and are fully biodegradable.

The encapsulation of curcumin in chitosan nanoparticles in the alginate-carrageenan matrices ensures the protection and stability of the compound while it moves in the digestive tract. This effect ensures the increased bioavailability of curcumin, and owing to the presence of alginate, also the prolonged release of the substance while it moves through the digestive tract. This was confirmed by a study of the release profile in vitro in PBS at pH = 7.4 (intestinal conditions, PBS is the phosphate-buffered saline) while using lyophilized particles. The study showed that as much as 95% of the fraction got through.

A simple investigation of the water solubility of nanoparticles based on chitosan proved that such a potential carrier increases the solubility of curcumin in the hydrophilic solution. The solubility of curcumin in water (0.6 μg/mL) after encapsulation into chitosan nanoparticles was improved up to 180 mg/mL. In these conditions, curcumin was well-dispersed and formed a homogeneous solution. What is more, the thus formed suspension had a naturally yellow color, which could indicate the inclusion of curcumin into dispersion of nanoparticles. A reference to that experiment was free curcumin added to water. It formed yellow, consolidated structures on the surface due to its limited solubility. In the above experiment, a much higher concentration of curcumin nanoparticles than of free curcumin was used. Thus, it can be supposed that the encapsulation of curcumin in chitosan nanoparticles can notably improve the release and dispersion of curcumin in the water solution, so it can increase its bioavailability during oral administration.

In this method (encapsulation of curcumin), the alginate-carrageenan matrix composition ratio is very important. Using SEM micrography (by applying the scanning electron microscope), it was shown that sodium alginate alone forms smoother and more spherical microparticles as compared with the alginate-carrageenan mixture. This effect is due to its greater ability of cross-linking in comparison with carrageenan. This allows one to obtain more stable particles than when using carrageenan alone, which forms rougher, less spherical, folded microparticles, of large pores and hollows. Therefore, while creating such a carrier, the proportions of the alginate are increased as compared with carrageenan. In this way, the physically formed particles are much more stable.

The release of curcumin was also analyzed depending on the pH, which is different in particular sections of the digestive tract. In acidic conditions (pH = 2.1), curcumin released from lyophilized microparticles ranged from 5% to 15% in all cases. This can suggest that in such an environment, curcumin could be absorbed from the digestive tract, and that it could be potentially protected against the acidic gastric juice. Whereas at pH = 7.4 (intestinal conditions), a small and gradual increase of curcumin release was observed in all the examined preparations. In comparison with the nanoparticles made of alginine alone, the release was of about 40% (38–39%), whereas using the alginate-carrageenan matrix (50:50 ratio) the release was as high as 96%. That is why the addition of carrageenan to the matrix is so important. After a 7 h release, a rapid degradation of curcumin was observed when alginine alone was used, whereas with the addition of carrageenan the degradation was spread over time and controlled.

The system of nanoparticles based on natural polymers increases the solubility and bioavailability of curcumin in gastrointestinal conditions. Thanks to the application of chitosan, the curcumin is dissolved in water much more effectively, whereas the use of the alginate-carrageenan matrix permits a longer and more controlled release of the active substance, owing to the ability to form hydrogel networks.

### 2.2. Silica Nanoparticles

Another form of nanoparticles are the silica nanoparticles, which are potentially one of the safest (non-toxic) forms of curcumin administration. Thanks to the appropriate structure and its hydrophilic surface, the release of curcumin is prolonged in time and the biocompatibility is increased. The synthesis of such particles is relatively easy and can be performed on a large scale. Silica nanoparticles are used not only to improve curcumin solubility, but also to increase the bioavailability of many medicinal substances [29].

**Figure 3:**
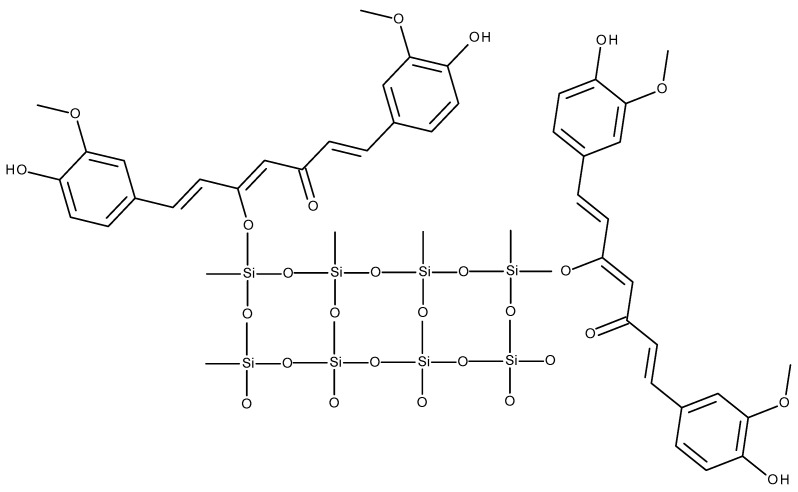
A postulated scheme of a curcumin-silica conjugate [30].

Curcumin is attached to the silica nanoparticles by the enolic hydroxyl group (Figure 3) with no effect on the basic functional group of curcumin—the diarylheptanoid one—which was observed in investigations by UV-Vis spectrophotometry and in the analysis using the Fourier-transform infrared spectroscopy (FTIR) [30].

A well-dispersed conjugate was obtained that was stable in an aqueous environment for as long as 2–3 weeks at room temperature. The solubility of curcumin increased by 71% in nanoparticles in comparison with free curcumin.

In the case of curcumin encapsulation in micelles from cetyltrimethylammonium bromide, and their subsequent coating with silica [31], using thermogravimetric analysis it was determined that about 40% of curcumin was packed into silica particles. A higher cytotoxicity of such a form was found as compared to unmodified curcumin [32].

The effect of cytotoxicity of curcumin nanoparticles was investigated on HeLa cells and on standard fibroblast cell lines. Already at a concentration of 2 µg/mL, a cytotoxic effect against HeLa cells was observed with a simultaneous lack of toxicity for healthy cells. When the concentration increased, the damage of healthy cells was also observed. At a concentration of nanoparticles of the order of 20 µg/mL, the compound destroyed not only HeLa cells (73%) but also the fibroblast cell line (40%), whereas at a 4-fold increased dose the fibroblast cell line was destroyed in 73%.

In the case of silica-based nanoparticles modified with hyaluronic acid, we observed an increased activation of caspase 3, one of those responsible for the development of apoptosis [33]. This higher effectiveness, as compared with free curcumin, can result from the increased endocytosis of nanoparticles, as it was observed based on a higher silica concentration in cancer cells SCC-25.

### 2.3. Single-Walled Carbon Nanotubes (SWCNTs)

Another example of nanostructures are the single-walled carbon nanotubes (SWCNTs), which are one of the allotropes of carbon. They are built of graphene sheets rolled up into thin tubes [34]. SWCNTs are an important class of artificial nanomaterials of exceptional physical and chemical properties which can potentially find new applications in nanomedicine as pharmaceutical auxiliary substances to form comprehensive drug delivery systems [35]. They are easy to take, internalized by the cells of mammals, and are also characterized by strong optical absorbance in a close infrared range of 0.8–1.4 μm (in which the tissue is transparent), which results in excessive local heating. Hence, they could also be used as a means of photothermal therapy used for ablation of cancer cells [36].

The single-walled carbon nanotubes were found to improve the bioavailability and solubility of curcumin, as well as its stability [37]. Owing to this technique, not only was the anticancer profile of curcumin improved, but also photothermal therapy was used. SWCNTs not only form the scaffolding for curcumin, but also serve as a means of thermal ablation, additionally inhibiting the cell growth (the study was performed on PC-3 cells, i.e., those of prostate cancer).

The length, the degree of aggregation, and the presence of functional groups of SWCNTs can be easily modified, which constitutes a very good agent of individualization and orientation of a given therapy. Hence, this method is frequently used for personalization and minimization of toxicity of the applied therapy.

One of the suggestions of functionalization was to deliver curcumin using SWCNTs by phosphatydylcholine (PC) and polyvinylpyrrolidone (PVP) [37]. The obtained preparation was evaluated as concerns its ability to load and release the curcumin, the protection of curcumin against degradation, and the increase of its solubility. The cytotoxicity of the preparation in vitro was examined, as well as the synergistic reinforcement of its anticancer activity by photothermal ablation of tumor cells using SWCNTs in PC-3 cancer cells to confirm the possibility of application of the delivery system in cancer therapies.

The effectiveness of curcumin packing into nanotubes was high, equal to 94%. This can be due to a large surface of SWCNTs, and to the molecular structure of curcumin, which contains two benzene rings and conjugated bonds, which makes easy absorption on SWCNTs possible, as well as its π-π complexation with the benzene ring.

The study of the formed complex showed that its anticancer properties were much stronger as compared with the free form. The framework of SWCNTs alone was also examined but it did not exhibit any such properties, nor did the low concentration with curcumin packed in (5 M). However, after exposition of SWNCTs-Cur (i.e., with curcumin) at a high concentration (15, 40 μM), the inhibition of PC-3 cells was observed, although this effect was dependent on both the concentration and the time of administration. Whereas, after the modification of SWNCTs-Cur by adding PC and PVP, the activity of curcumin was already observed at a low concentration.

The increase of curcumin solubility in comparison with the free fraction was confirmed by the spectrophotometric method, and thanks to the addition of PC and PVP, the SWCNTs were even better dispersed. At the same time, it was observed that only 5% of curcumin was hydrolyzed and metabolized, which confirms the reduced biodegradation.

However, some studies indicate that the physicochemical properties of SWCNTs, including their highly hydrophobic surface, insolubility in water solutions, and the pollution with metals used in the production process, can lead to their toxicity and weak biocompatibility, so careful optimization of physicochemical parameters is needed to minimize the toxicity of carbon nanotubes, which can lead to biomedical applications [38].

### 2.4. Nanoparticles of the Poly Lactide-Glycolide (PLGA) Polymer

The PLGA nanoparticles are yet another form of curcumin which showed greater solubility in comparison with the free fraction. PLGA is a widely used biocompatible polymer which facilitates a slow and extended release of drugs that are packed in them [39].

The applied formulation was formed as a result of using two combinations: poly (lactide-co-glycolide) in the 50:50 or 75:25 ratio (lactide:glycolide) [40]. Since the degradation rate of PLGA depends on the lactide:glycolide ratio, it was expected that the kinetics of drug release would be dependent on the copolymer ratio. At the nanoparticle ratio of 50:50, much smaller particle dimensions were observed with a higher effectiveness of curcumin packing, as well as its much more rapid release (even with a 9-fold increase of bioavailability). In the studies on cervical cancer cells (HeLA), it was observed that nanocurcumin at 50:50 exhibited a much stronger anticancer activity in comparison with the 75:25 formulation and free curcumin. Moreover, an increase of induced caspase activation was described. It was also observed that the inhibition of NF-kB by nanocurcumin at 50:50 is much higher than in the case of curcumin in the free fraction.

The kinetics of release of curcumin particles from the PLGA matrix was characterized by a fast initial release which extended up to a week. The initial rapid release can be due to curcumin which was present on the surface or was weakly packed in the polymer matrix, whereas a slow and continuous release can be ascribed to the diffusion of curcumin localized in the core of PLGA nanoparticles. It should be noted that nanocurcumin at 50:50 showed a faster release, which was probably due to the presence of a higher glycolide content in this proportion, which in turn conditions quicker degradation than in the 75:25 matrix.

### 2.5. Nanosuspensions

Nanosuspensions are a two-phase form in which the dispersed molecules do not exceed the dimensions of 1 µm [41]. This form of products has recently gained great interest in the pharmaceutical industry owing to its ability to enhance the solubility of weakly dispersed drugs [42]. A new form of curcumin dosage as a nanosuspension (CUR-NS) was prepared by high-pressure homogenization (HPH) [43]. Curcumin in this form showed a much-increased water solubility and was characterized by an even 10-fold faster dissolution in comparison with curcumin in the free form.

The investigation of curcumin solubility in the new form was performed by the paddle method according to specifications of Chinese Pharmacopoeia for weakly soluble drugs. A much higher (even 600-fold) solubility of the compound was found. A high adhesion of CUR-NS was also observed as well as increased permeability through the cell membranes, which led to higher cytotoxicity. A higher level of bonding and capture of the nanosuspension could result from the fact that during its production, sodium deoxycholate and soy lecithin were used as stabilizers. The nanosuspension, that is a new form of curcumin, was dedicated for intravenous drug administration. While arranging/designing this form, it should be remembered that the formulation must not cause local irritation of veins or vein inflammation, and that it should be characterized by a low thrombocyte hemolysis index. The results of the performed investigations on the effectiveness and safety of nanosuspensions suggest that CUR-NS can be a promising strategy to use in cancer therapies by intravenous delivery.

## 3. Complexes with Cyclodextrins (CDC)

**Figure 4:**
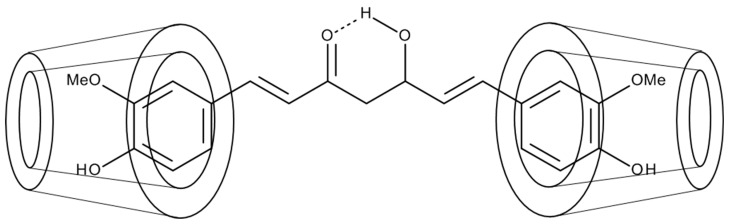
A proposed structure of the curcumin-cyclodextrin inclusion complex.

Another formula enhancing the bioavailability of curcumin is its combination with cyclodextrins (CDC). Cyclodextrins are cyclic oligosaccharides forming structures of a hydrophilic external surface and the lipophilic center. The introduction of lipophilic drugs or their groupings into the central niche of a cyclodextrin permits the formation of a much more soluble complex because the CDC coat is of hydrophilic character (Figure 4) [44].

There are several methods of formation of such a curcumin-cyclodextrin complex (β-CD). One of them consists in mixing the components in the mass ratio of 1:4 (curcumin:cyclodextrin), followed by dissolution in water and subsequent sonification and shaking [45]. The complex with β-cyclodextrin not only improved the solubility of curcumin, but also exhibited better antioxidation properties in in vitro tests (DPPH and ABTS tests) and better anticancer properties, as examined on the A375 cell line [45]. The obtained complexes probably intensified the apoptosis of cancer cells.

In another study [46], it was shown that the obtained complexes were characterized by a greater dissolution rate as compared with the pure substance, and they had a better antioxidation profile than in an earlier described investigation.

## 4. Polymorphic Forms

The crystal active substances (API—Active Pharmaceutical Ingredient) can occur in several polymorphic forms which differ in crystallographic parameters. The polymorphic forms can differ in solubility (including water solubility) and increased the bioavailability by even 2–4 times. This creates new possibilities and new challenges, in particular in the generic drugs market. When introducing a new polymorphic form of a drug on the market in place of a previous one, a key role is played by confirmation of its stability and bioequivalence. This is because the new polymorphic form of the same substance may be characterized by a longer half-life, greater solubility and/or bioavailability, and an extended-release profile [47]. These changes can affect drug formulation, effectiveness, and dose.

**Figure 5:**
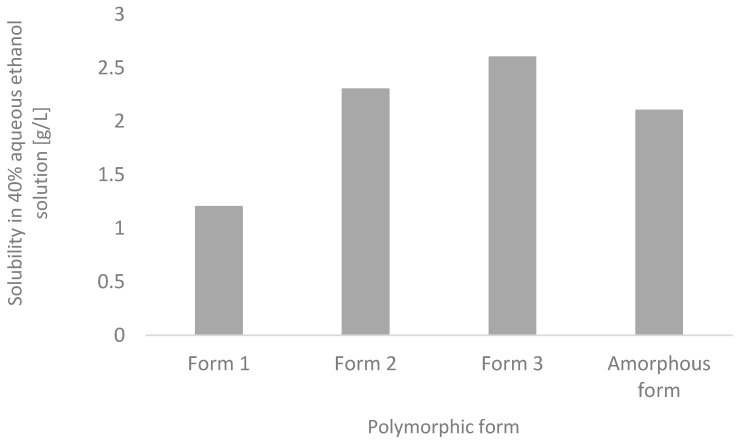
Solubility of different varieties of curcumin in 40% aqueous ethanol solution [48].

So far, three polymorphic forms for curcumin have been described, and one amorphous form without spatial arrangement [48]. The amorphous form of curcumin can be obtained by cooling the melted substance at room temperature or at a temperature of about 10 °C. Commercially available curcumin occurs in the polymorphic form 1. This form is in the space group P2/n (Z = 4, Z0 = 1). The polymorphic form 2, which is situated in the space group Pca21 (Z = 8, Z0 = 2), can be obtained by co-crystallization from 4-hydroxypyridine in ethanol at room temperature, by crystallization from DMSO at room temperature, or by crystallization from the saturated curcumin solution from ethanol kept at 10 °C for two days. The polymorphic form 3, which is in the space group Pbca (Z = 8, Z0 = 1), can be obtained by crystallization from 4,6-dihydroxy-5-nitropyrimidine as a co-former. All the above-described polymorphic forms contain a curcumin molecule in the form of a keto-enolic tautomer. In form 1, the curcumin molecule has a curved, slightly twisted conformation. On both sides of the molecule, the hydrogen bonds are formed between phenolic groups of curcumin with subsequent molecules which combine with the fourth molecule of curcumin, forming a macrocyclic ring of hydrogen bonds. Forms 2 and 3 do not create such a ring because they have a linear molecular shape. According to a study on the stability of polymorphic forms by crystallization from a suspension in 40% ethanol at room temperature, it was shown that during 24 h, form 2 and the amorphous form are transformed into form 1. It was assumed that there is an opposite correlation between the stability of the polymorphic form of a crystal and its solubility, which means that the less stable the polymorph is, the faster and the better it will dissolve in water. This dependence is confirmed by investigations into the solubility rate in 40% ethanol, where form 2 has a 3.1-fold higher solubility rate as compared with form 1. Figure 5 presents data on the solubility of different varieties of curcumin in 40% aqueous ethanol solution [48].

## 5. Cocrystals of Curcumin

Cocrystals are the heteromeric products of co-crystallization. This process makes it possible to obtain the substances of new physicochemical properties such as changed solubility, stability, or mechanical properties. That is why in recent years, a growing interest in this kind of formulation has been observed in pharmacy. Pharmaceutical cocrystals consist of the active substance (API) and a co-component which can be both a therapeutically active molecule (drug–drug cocrystal) and an inert molecule [49]. Using this technique, one can modify the properties of compounds (such as solubility, stability, or permeability through biological membranes) which are of key importance to enhance the bioavailability of the medicinal substance.

The increase of water solubility of a biologically active substance can be obtained by its co-crystallization with another substance which is several times more soluble. Both kinds of molecules are combined by weak hydrogen bonds which are broken in the aqueous medium after a short time (from 1 min to several hours). The co-former which is better-soluble in water will extract to the solution from the crystal structure, leaving the molecules of the active substance floating in the solution, connected only by loose and weak intermolecular interactions, forming an amorphous form of a higher energy. That is because the transfer of the co-former to the solution is faster than the aggregation of the molecules of the active substance. This amorphous state of the molecules of the active substance results in a considerable increase of its concentration in the solution and the formation of a supersaturated system. This state is known as “Spring”. However, after some time, the active substance begins to aggregate again. The formation of the supersaturated solution (“Spring”) can be used to improve the absorption of the active substance, which, however, should be kept in an amorphous state for about 120–300 min. The prolongation of the “Spring” state can be obtained by using the appropriate pharmaceutical auxiliaries, which inhibit the nucleation and/or growth of cocrystals, and such substances are called “precipitation inhibitors” or “parachute”. After some time, the high-energy solution with the amorphous phase will be transformed into a metastable polymorphic form of the drug, also of higher solubility, and will eventually reach a stable form according to Ostwald’s dilution law.

At present, some drugs in the cocrystal form are already available on the market, e.g., Entresto made by Novartis, used for the treatment of heart failure. This drug contains a composition of two medicinal substances: sacubitril and valsartan.

The main advantages of using co-crystallization for the modification of the properties of medicinal substances are as follows:

1. The molecular structure of the substance remains unchanged while its properties are affected by the co-former.
2. Thanks to understanding the principles of crystal engineering, the structure of a cocrystal can be controlled.
3. Thanks to the library of available synthons and co-formers, it is possible to select the required functional properties for a large number of APIs.
4. The form of a cocrystal, in comparison to salt, can also be obtained for the substances which do not undergo ionization, or which have sensitive functional groups that can undergo transformation when affected by strong acids and bases.

As concerns curcumin, studies on cocrystals of this substance have also been recently performed. They are first investigated to improve the water solubility of curcumin and hence to enhance its bioavailability. The analysis of the structure of chemical compounds that form cocrystals with curcumin can allow the researchers to better understand which interactions are responsible for the formation of a crystal structure. Table 1 presents the summary of the hitherto published studies on the cocrystals of curcumin

## 6. Conclusions

All the methods, techniques, or modes of action presented in this article considerably help to improve the solubility of curcumin, which will enable the utilization of its significant medicinal potential originating from its activity for key molecular purposes. The increase of its absorption from the digestive tract due to better solubility and enhanced transport to the tissues will permit the researchers to obtain optimum therapeutic values of the substances, which in turn will help to achieve more effective results with lower doses of drugs applied. Presently, numerous studies are being performed aiming to determine the most effective modes of drug delivery as well as the formulations (e.g., microencapsulation, nanosuspensions, or complexes with cycloserine). It is very popular to combine curcumin with black pepper, which according to the performed studies increases the absorption and bioavailability of curcumin up to 20-fold. The currently performed investigations on cocrystals of curcumin are also very promising. An important aspect is also the possibility of the occurrence of crystal active substances in several polymorphic forms, which can differ in solubility (in water) and bioavailability that can be several times greater. This creates new perspectives and new challenges in the market of generic drugs.

An interesting issue for future research is also looking for methods to improve the solubility and bioavailability of turmeric extract rich in curcuminoids. For now, the most common solution used in food supplements is the use of piperine. Piperine acts as a bioenhancer and decreases curcumin metabolism in an organism. However, the encapsulation of extracts in polysaccharide nano-capsules may also be applied.

## Figures and Tables

**Table 1:** Summary of information about selected curcumin cocrystals.

Co-Former	Preparation Method	Molar Ratio	Intermolecular Interactions	Change of Solubility	Ref.
Resorcinol	Hand fragmentation (liquid-assisted) of particular solid components for 30 min in a mortar after adding 5–6 drops of ethanol	1:1	Intermolecular hydrogen bond between phenolic group of resorcinol and carbonyl group of curcumin Alternatively, weak C-H···O interaction between methoxyl group of resorcinol and C-H···π interaction	Increase of solubility in ethanolic solution (4.72-fold)	[50]
Pyrogallol	Hand fragmentation (liquid-assisted) of particular solid components for 30 min in a mortar after adding 5–6 drops of EtOH	1:1	Intermolecular hydrogen bond between phenolic group of pyrogallol and carbonyl group of curcumin Formation of a trimer between two molecules of curcumin and one molecule of pyrogallol by O-H···O bonds between phenolic groups of two curcumin molecules and oxygen acceptor of pyrogallol	Increase of solubility in ethanol–water solution (11.76-fold)	[50]
Phloroglucinol	Dissolution of curcumin and phloroglucinol in organic solvent removed in vacuo using a water evaporator, drying for 3 h at 40 °C	1:1	No data	Reduction of solubility	[51]
4,4′-bipyridine-N,N′-dioxide	Suspension of curcumin and 4,4′-bipyridine-N,N′-dioxide (BPNO) in ethanol–acetone mixture, stirring at room temperature. After heating the suspension at 60 °C for 72 h, left to evaporate for 24 h.	1:1	Hydrogen bond between oxygen of 4,4′-bipyridine-N,N′-dioxide and the -OH group of phenolic group of curcumin	No data	[52]
Hydroxyquinol	Heating up to 130 °C and dissolution in equimolar acetone-toluene or ethyl acetate-toluene mixture	1:1	Strong hydrogen bond between -OH group of hydroxyquinol and β-diketo of curcumin	Increase of solubility from acetone-toluene mixture 1.6-fold, and from ethyl acetate-toluene mixture 1.6-fold.	[53]
	Heating up to 135 °C and dissolution in 10 mL of equimolar acetone-toluene or ethyl acetate-toluene mixture at sonification	1:2		Increase of solubility from acetone-toluene mixture 2.8-fold, and from ethyl acetate-toluene mixture 3-fold.	
Methylparaben	Wet grinding with ethanol	1:1	Intermolecular hydrogen bonds between hydroxyl group of methylparaben and β-diketo of curcumin	No data	[54]
2-aminobenzimidazole	Grinding of curcumin-2-aminobenzimidazole mixture by adding methylisobutylketone dropwise in a ball mill	1:1	No data	Increase of solubility in aqueous ethanolic solution: IDR 0.023 ((mg/cm^2^)/min) ^1^	[55]
Nicotinamide	Grinding of curcumin-nicotinamide mixture by adding ethyl acetate dropwise in a ball mill	1:1	No data	Increase of solubility in aqueous ethanolic solution: IDR 0.040 ((mg/cm^2^)/min) ^1^	[55]
L-lysine	Grinding of curcumin-L-lysine mixture in a ball mill	1:1	No data	Increase of solubility in aqueous ethanolic solution: IDR 0.029 ((mg/cm^2^)/min) ^1^	[55]
Piperazine	Grinding of suspension in acetonitrile for 6 and 24 h (for polymorphic forms 1 and 2, respectively)	1:1	No data	No data	[55]
	Grinding of suspension in methanol for 4 h	2:1			
Isonicotinamide	Dissolution of equimolar mixture in n-propyl acetate or ethyl acetate, then slow evaporation	1:2	No data	No data	[55]
Sodium naproxen	Grinding the suspension in acetone	1:1	No data	No data	[55]
Piperidine	Grinding the suspension in acetonitrile	1:1	No data	No data	[55]
Sodium ibuprofen	Sonification of curcumin-sodium ibuprofen mixture in acetone for 15 min	1:1	No data	No data	[55]

^1^ IDR—Intrinsic dissolution rate is the dissolution rate of a defined surface of a pure substance at constant temperature and pH, for the polymorphic variety 1 of curcumin IDR = 0.00796 ((mg/cm^2^)/min).

## Data Availability

Data sharing not applicable.
